# A Modified Azimuth Weighting Method in a Two-Step Process Approach for Sliding Spotlight Data Processing

**DOI:** 10.3390/s17020220

**Published:** 2017-01-24

**Authors:** Feng Xiao, Ze-gang Ding, Bin Xiong, Teng Long

**Affiliations:** Beijing Key Laboratory of Embedded Real-time Information Processing Technology, School of Information and Electronics, Beijing Institute of Technology, Beijing 100081, China; 312010349@bit.edu.cn (F.X.); xb1120110409@126.com (B.X.); longteng@bit.edu.cn (T.L.)

**Keywords:** weighting method, two-step processing approach, sliding spotlight, synthetic aperture radar (SAR)

## Abstract

Low sidelobes are important and essential in all SAR (Synthetic Aperture Radar) images, regardless of the imaging mode, for fewer artificial targets. For strip-map mode all targets overlap in frequency, which is convenient to suppress sidelobes. However, weighting requires total overlap in the time or frequency domain, which a sliding spotlight signal could not satisfy. Furthermore, the wavelength cannot be regarded as a constant value under the condition of a wideband chirp signal, which leads to the variation of the Doppler bandwidth along with the range frequency. In this article, an azimuth weighting method is proposed that considers the influence of a wideband based on a two-step algorithm. The computer simulation is given to verify the presented method.

## 1. Introduction

By means of properly steering a radar antenna in the along-track direction, SAR enlarges its applications in both civil and military domains, especially in the sliding spotlight mode [1,2,3,4,5,6]. Compared to the traditional spotlight mode, the sliding spotlight mode could enable wider coverage in the azimuth direction. Just as in the spotlight case, the key problem with the sliding spotlight focus processing is in the aliasing of the azimuth echo.

However, the shift of the Doppler centroid introduced by beam rotation will cause the Doppler bandwidth of the scenario to be larger than the pulse repetition frequency (PRF) [7,8,9]. Additionally, under the condition of a wideband signal, the variation of residual Doppler bandwidth along the range frequency must be taken into consideration while conducting the weighting operation.

The classical two-step imaging algorithm is introduced to overcome the azimuth spectrum aliasing [7,10,11,12], but the azimuth residual Doppler bandwidth is not carefully analyzed and the sidelobe suppression is not considered, especially for high-resolution imaging. Sub-aperture algorithms are another way to deal with the azimuth spectrum aliasing [6,13], but it is difficult to conduct an azimuth weighting operation since the different azimuth targets share different time support domains and different frequency support domains. Based on the azimuth frequency de-ramping principle, a novel processing approach is proposed [14,15], with the disadvantage of more FFT (Fast Fourier Transform) and IFFT (Inverse Fast Fourier Transform) operations and lower efficiency. Furthermore, a new algorithm named band azimuth scaling (BAS) is proposed for both TOPS (Terrain Observation by Progressive Scans) and sliding spotlight data imaging [16,17,18,19,20]. However, the BAS algorithm needs a sub-aperture combination. Based on a frequency scaling algorithm (FSA) and spectral analysis (SPECAN), a highly accurate algorithm for spotlight data is proposed [8,21], whose azimuth weighting is not considered and needs sub-aperture processing. Back-projection (BP) and fast BP [22,23,24,25] are other ways to focus SAR echoes precisely without considering the work mode, but the sidelobe suppression of several work modes, such as the sliding spotlight mode and TOPS mode, need to be considered. Additionally, the computational load may be further improved. From former analysis, it is clearly that the azimuth weighting method in the sliding spotlight mode is not carefully discussed. Therefore, based on a two-step algorithm, an azimuth weighting method is proposed, which is suitable for high-resolution data processing.

The paper is organized as follows: In the second section, the signal model is established, which includes the geometry and echo model. In the third section, based on curved geometry, the algorithm of weighting windows in frequency is declared. In addition, the comparison of the former weighting method and the proposed method is illuminated. In the fourth section, the simulation results validate the presented analysis. Conclusions are drawn in the fifth section.

## 2. Signal Characteristics Analysis

### 2.1. Imaging Geometry

**Figure 1:**
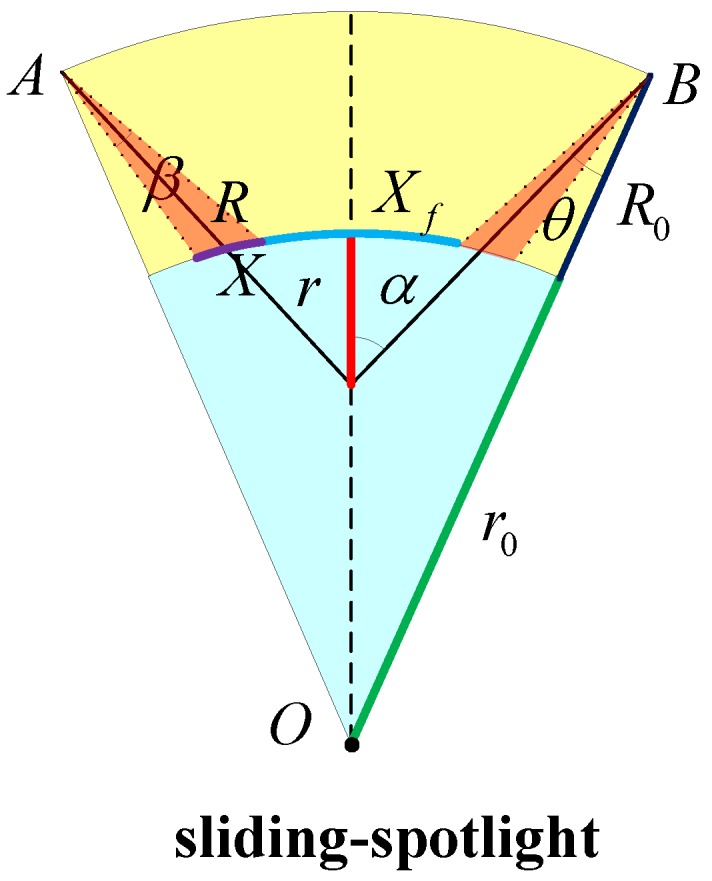
Space-borne sliding-spotlight mode planar imaging curved Earth geometry. A and B are the orbit start and end. The orange region represents the beam. The blue region represents the Earth’s interior.

The planar curved imaging geometry of the sliding-spotlight mode is shown in Figure 1, which indicates that the azimuth beam is steered from fore to aft at a constant rotation rate as:

(1) $$\omega_{r}=\frac{d\theta }{dT},$$

where θ is the instantaneous squint angle and T is the whole acquisition interval, and R and r are the slant ranges from the flight path to the scene center and the virtual rotation center to the scene center, respectively. O is the geocenter, β is the azimuth beam width, X≈βR is the width in azimuth direction, and $X_{f}$ represents the width of the scene. α is the rotation angle.

For azimuth beam scanning at a constant rotation rate leading to $\omega_{r}=\theta /T$, the steering factor A is defined as [14]:

(2) $$A=\frac{r}{R+r},$$

Assuming that the azimuth beam steering from fore to aft is positive, the azimuth resolution of the sliding-spotlight mode can be approximately computed as [13]:

(3) $$\rho_{AZ}=\frac{D_{a}}{2}\cdot \frac{V_{g}}{V_{s}}\cdot \gamma_{w,a}\cdot A=\frac{D_{a}}{2}\cdot A,$$

where $V_{s}$ and $V_{g}$ are the physical velocity of the SAR sensor and the footprint velocity, respectively without taking into account the azimuth beam steering. $D_{a}$ is the antenna length and $\gamma_{w,a}$ is the impulse response width broadening factor due to an azimuth processing window. The approximation $V_{g}/V_{s}\gamma_{w,a}\approx 1$ is reasonable in the majority case.

### 2.2. Properties of the Echo Signal

If the transmitter illuminates the target scene with a baseband chirp signal p(t) and a point target locates at $\left(x_{0},R\right)$, the echo of transmitted pulse can be expressed as:

(4) $$\begin{array}{cc} s\left(\tau ;t_{a},x\right) & =rect\left[\frac{\tau -\frac{2R\left(t_{a}\right)}{c}}{T_{p}}\right]\cdot \exp\left[-j\frac{4\pi R\left(t_{a}\right)}{\lambda }\right]\cdot \exp\left[j\pi K_{r}{\left(\tau -\frac{2R\left(t_{a}\right)}{c}\right)}^{2}\right]\\ & \cdot \;rect\left[\frac{V_{f}t_{a}-x_{0}}{X}\right]\cdot rect\left[\frac{x}{X_{f}}\right] \end{array},$$

where $\lambda =c/f_{0}$ is the wavelength, $K_{r}=B/T_{p}$ is the FM (frequency modulation) rate. $V_{f}$ is the footprint velocity taking into account azimuth beam steering. τ and $t_{a}$ are fast time and slow time variables, respectively. The azimuth signal of the echo is aliasing which cannot be weighted now. Based on rectilinear imaging geometry of sliding-spotlight mode, $R\left(t_{a}\right)$ can be written as:

(5) $$R\left(t_{a}\right)\approx \sqrt{R_{0}^{2}+V_{r}^{2}\cdot {t_{a}}^{2}},$$

where $V_{r}\approx \sqrt{V_{g}\cdot V_{s}}$. $V_{r}$ is usually used for the imaging focus and $V_{s}$ is used for calculating the Doppler bandwidth.

## 3. Precious Azimuth Weighting Method in a Two-Step Algorithm

**Figure 2:**
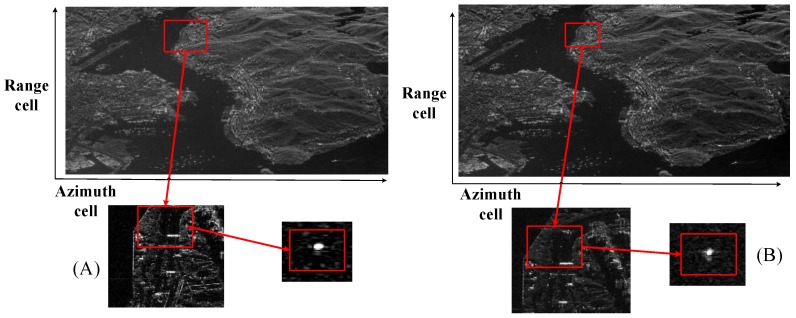
C-band VV-polarized image of Hong Kong obtained by the GF-3 system in sliding spotlight mode in 2016. (**A**) Using a weighting operation, and (**B**) without a weighting operation. Azimuth resolution is about 1 m. The extension of the area is of about 8 km × 10 km.

Generally speaking, the sidelobes after pulse compression are not as low as expected, which leads to shade weak objects. Thus, a weighting technique is necessary in the image processing. The comparison between employing a weighting method and without weighting processing in the sliding-spotlight mode is shown in Figure 2.

Real data from GF-3 is shown as Figure 2. The quantization methods are same. It clearly shows the weighting effects in the SAR image. The sidelobes of the strong target in the right picture are stronger than the surrounding targets and the weak targets are buried. Compared to the right figure, which is not using a weighting operation, the sidelobes of the left picture are lower.

In this section, based on a deramping method, the total weighting method is proposed in the first part, the comparison of the azimuth weighting processing in the range time domain [26] and proposed method are analyzed in the second part.

### 3.1. Azimuth Preprocessing and Weighting Method

Considering the rotation of the azimuth beam under the curved Earth geometry, the instantaneous range from the satellite to the virtual rotation center $R_{c}\left(t_{a}\right)$ can be expressed as:

(6) $$\begin{array}{cc} R_{c}\left(t_{a}\right) & =\sqrt{{\left(R+r\right)}^{2}+V_{s}^{2}\cdot {t_{a}}^{2}}\\ & \approx \left(R+r\right)+\frac{V_{s}^{2}\cdot {t_{a}}^{2}}{2\left(R+r\right)}+\frac{V_{s}^{4}\cdot {t_{a}}^{4}}{8{\left(R+r\right)}^{3}} \end{array}.$$

Next, the $f_{dc}$ in the virtual point can be calculated via $f_{dc\_rot}\left(t_{a}\right)=-2/\lambda \cdot dR_{c}\left(t_{a}\right)/dt_{a}$.

Then the azimuth variation rate $f_{rot}$ causing by azimuth beam rotation can be expressed as:

(7) $$f_{rot}\left(t_{a}\right)=\frac{df_{dc\_rot}\left(t_{a}\right)}{dt_{a}}=-\frac{2}{\lambda }\cdot \left[\frac{V_{s}^{2}}{\left(R+r\right)}+\frac{3V_{s}^{4}\cdot {t_{a}}^{2}}{2{\left(R+r\right)}^{3}}\right].$$

Under the typical low orbit parameter and carrier frequency is C band, $f_{rot}$ can be expressed approximately as:

(8) $$\begin{array}{cc} f_{rot} & \approx -\frac{2}{\lambda }\cdot \frac{V_{s}^{2}}{\left(R+r\right)}\\ & =-\frac{2V_{s}}{\lambda }\cdot \frac{2\theta }{T}\\ & =-\frac{2V_{r}}{\lambda }\cdot \frac{2\theta_{r}}{T} \end{array},$$

where $\theta_{r}$ is equivalent instantaneous squint angle. Thus, the azimuth bandwidth caused by the beam rotation can be expressed as:

(9) $$\begin{array}{cc} B_{rot} & =abs\left[f_{rot}\cdot T\right]\\ & =\frac{2V_{s}}{\lambda }\cdot 2\theta \\ & =\frac{2V_{r}}{\lambda }\cdot 2\theta_{r} \end{array}.$$

Compared with the strip-map mode, the azimuth original bandwidth caused by the beam width is:

(10) $$B_{beam}=\frac{2V_{s}}{\lambda }\cdot \beta .$$

Finally, the azimuth total Doppler bandwidth can be expressed as:

(11) $$B_{total}=B_{rot}+B_{beam}.$$

**Figure 3:**
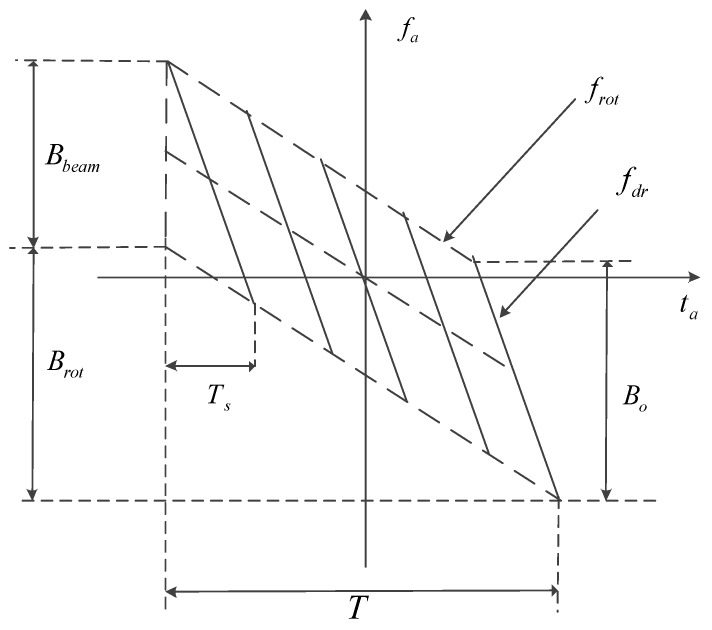
Doppler time-frequency relationship in the space-borne sliding-spotlight mode.

Figure 3 shows the Doppler time-frequency relationship.

Where $f_{dr}=2V_{r}^{2}/\left(\lambda \cdot R_{0}\right)$ is the azimuth FM rate. $T_{s}$ represents synthetic aperture time and $B_{o}$ is the Doppler course of a single point, respectively.

In general, the total bandwidth is larger than the PRF in the sliding spotlight mode. In order to overcome the aliasing of the azimuth echo in the frequency domain, azimuth convolution processing is conducted, which is the key point of azimuth preprocessing. The quadratic phase signal is expressed as:

(12) $$\begin{array}{cc} g\left(t_{a}\right) & =\exp\left(-j\pi \cdot f_{rot}\cdot {t_{a}}^{2}\right)\\ & =exp\left(j\pi \cdot \frac{2V_{s}}{\lambda }\cdot \frac{2\theta }{T}\cdot {t_{a}}^{2}\right)\\ & =exp\left(j\pi \cdot \frac{2V_{s}^{2}}{\lambda \left(R+r\right)}\cdot {t_{a}}^{2}\right) \end{array},$$

While conducting the azimuth weighting processing in range time domain, the azimuth echo preprocessing can be accomplished by employing:

(13) $$\begin{array}{c} c\left(t_{\tau },t_{a}\right)\\ =S\left(t_{\tau },t_{a}\right)⊗g\left(t_{a}\right)\\ =\int S\left(t_{\tau },z\right)\cdot g\left(t_{a}-z\right)dz \end{array}.$$

Substituting Equation (12) into Equation (13), Equation (13) can be rewritten as:

(14) $$c\left(t_{\tau },t_{a}\right)=\underset{\begin{array}{l} phase\\ compensation \end{array}}{\underset{︸}{\exp\left[j2\pi \cdot \frac{V_{s}^{2}t_{a}^{2}}{\lambda \left(R+r\right)}\right]}}\underset{FT}{\underset{︸}{\cdot \int \underset{dechirp}{\underset{︸}{S\left(f_{\tau },t_{a}\right)\cdot \exp\left[j2\pi \cdot \frac{V_{s}^{2}\cdot z^{2}}{\lambda \left(R+r\right)}\right]}}\cdot \exp\left[-j2\pi \cdot \frac{2V_{s}^{2}\cdot z\cdot t_{a}}{\lambda \left(R+r\right)}\right]dz}}.$$

**Figure 4:**
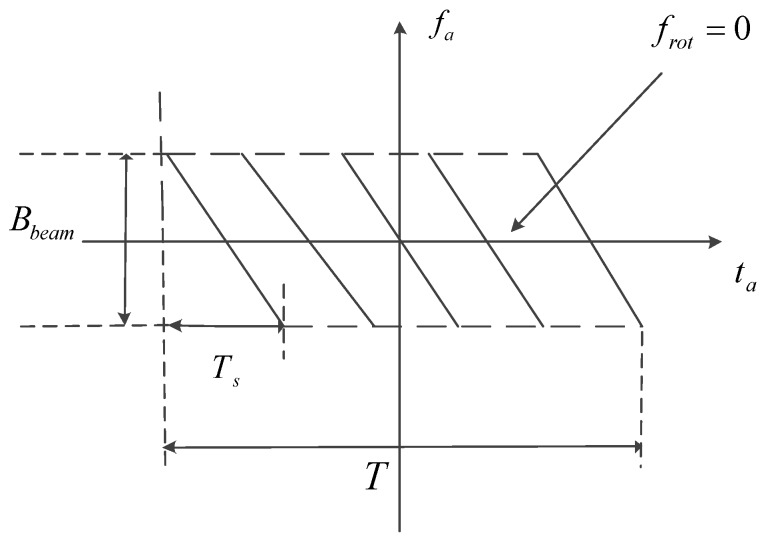
The Doppler time-frequency relationship after dechirp processing, the signal totally overlaps in the support domain which can be weighted. The time-frequency relationship is under the condition of the narrowband signal.

From Equation (14), the azimuth convolution processing includes three parts: dechirp processing, Fourier transform, and phase compensation processing. Figure 4 shows the azimuth time-frequency relationship after dechirp processing.

Note that the wavelength is defined for a single-frequency signal but, for the chirp signal, the wavelength is not a constant value, so it can be expressed approximately as $\lambda \approx c/f_{0}$, and the azimuth convolution can be carried out both in the range time domain and range frequency domain under the condition of the narrowband signal, while under the condition of the wideband signal, the convolution must be carried out in range frequency domain [7] and:

(15) $$\lambda_{equal}\left(f_{\tau }\right)=\frac{c}{f_{0}+f_{\tau }}$$

The parameter $\lambda_{equal}\left(f_{\tau }\right)$ is defined in the range frequency domain, which is related with $f_{\tau }$. This parameter indicates all wavelengths in the chirp signal and can be used in the range frequency domain to describe the wavelength for the chirp signal precisely instead of in the range time domain. The error analysis of the azimuth weighting method in [26] is analyzed in the next part.

Conducting the azimuth weighting processing in the range frequency domain, the azimuth echo preprocessing by convolution can be written as:

(16) $$\begin{array}{cc} c\left(f_{\tau },t_{a}\right) & =S\left(f_{\tau },t_{a}\right)⊗g\left(t_{a}\right)\\ & =\int S\left(f_{\tau },z\right)\cdot g\left(t_{a}-z\right)dz \end{array}.$$

Combining Equations (14)–(16), then:

(17) $$\begin{array}{cc} c\left(f_{\tau },t_{a}\right) & =\underset{\begin{array}{l} phase\\ compensation \end{array}}{\underset{︸}{\exp\left[j2\pi \cdot \frac{V_{s}^{2}\cdot t_{a}^{2}}{\frac{c}{\left(f_{0}+f_{\tau }\right)}\cdot \left(R+r\right)}\right]}}\\ & \underset{FT}{\underset{︸}{\cdot \int \underset{dechirp}{\underset{︸}{S\left(f_{\tau },t_{a}\right)\cdot \exp\left[j2\pi \cdot \frac{V_{s}^{2}\cdot z^{2}}{\frac{c}{\left(f_{0}+f_{\tau }\right)}\cdot \left(R+r\right)}\right]}}\cdot \exp\left[-j2\pi \cdot \frac{2V_{s}^{2}\cdot z\cdot t_{a}}{\frac{c}{\left(f_{0}+f_{\tau }\right)}\cdot \left(R+r\right)}\right]dz}} \end{array}.$$

is derived. Implementing Equation (17) in the discrete domain, it can be written as:

(18) $$\begin{array}{cc} c\left(f_{\tau },\Delta x_{1}\right) & =\exp\left[j2\pi \cdot \frac{{\left(n\cdot \Delta x_{2}\right)}^{2}}{\frac{c}{\left(f_{0}+f_{\tau }\right)}\cdot \left(R+r\right)}\right]\\ & \cdot \sum_{i=-\frac{N_{a}}{2}}^{i=\frac{N_{a}}{2}}S\left(f_{\tau },\Delta x_{1}\right)\cdot \exp\left[j2\pi \cdot \frac{{\left(i\cdot \Delta x_{3}\right)}^{2}}{\frac{c}{\left(f_{0}+f_{\tau }\right)}\cdot \left(R+r\right)}\right]\cdot \exp\left[-j2\pi \frac{2\cdot \Delta x_{2}\cdot \Delta x_{3}}{\frac{c}{\left(f_{0}+f_{\tau }\right)}\cdot \left(R+r\right)}\right] \end{array},$$

where:

(19) $$\begin{array}{l} \Delta x_{1}=\frac{V_{r}}{PRF}\\ \Delta x_{2}=\frac{V_{s}}{PRF_{up}}\\ \Delta x_{3}=\frac{V_{s}}{PRF} \end{array},$$

then Equation (18) can be expressed as:

(20) $$\begin{array}{cc} c\left(n\Delta x^{\prime \prime }\right) & =\exp\left[j2\pi \cdot \frac{{\left(n\cdot \Delta x_{2}\right)}^{2}}{\frac{c}{\left(f_{0}+f_{\tau }\right)}\cdot \left(R+r\right)}\right]\\ & \cdot \sum_{i=-\frac{P}{2}}^{i=\frac{P}{2}}S\left(f_{\tau },\Delta x_{1}\right)\cdot \exp\left[j2\pi \cdot \frac{{\left(i\cdot \Delta x_{3}\right)}^{2}}{\frac{c}{\left(f_{0}+f_{\tau }\right)}\cdot \left(R+r\right)}\right]\cdot \exp\left[-j2\pi \cdot \frac{2\cdot \Delta x_{2}\cdot \Delta x_{3}}{\frac{c}{\left(f_{0}+f_{\tau }\right)}\cdot \left(R+r\right)}\right]\\ & \approx \exp\left[j2\pi \cdot \frac{{\left(n\cdot \Delta x_{2}\right)}^{2}}{\frac{c}{\left(f_{0}+f_{\tau }\right)}\cdot \left(R+r\right)}\right]\cdot DFT\left\{S\left(f_{\tau },\Delta x_{1}\right)\cdot \exp\left[j2\pi \cdot \frac{{\left(i\cdot \Delta x_{3}\right)}^{2}}{\frac{c}{\left(f_{0}+f_{\tau }\right)}\cdot \left(R+r\right)}\right]\right\} \end{array},$$

which means using FFT is a good choice to improve algorithm efficiency. After dechirp processing the signal totally overlaps, so the weighting operation can be carried out at this time. The total residual bandwidth can be written as:

(21) $$B_{ar}\approx \frac{2V_{s}\cdot \beta }{c}\cdot \left(f_{0}+f_{r}\right).$$

The azimuth weighting method in [26] is carried out in the range time domain, which is accurate in the high-resolution imaging mode, but this method causes significant errors while the bandwidth is about an order of the carrier frequency. It is impossible to consider the effect by the range frequency if the azimuth preprocessing is conducted in the range time domain. Only by updating the $f_{r}$ in the range frequency domain is the azimuth window is precisely produced. A detailed analysis and more experiments illustrate this below.

We further remark that the parameter design of PRF must satisfy $PRF>2V_{s}/\left(c/f_{0}\right)\cdot \beta +V_{s}/\rho_{r}\cdot \beta$, where $\rho_{r}=c/\left(2B_{r}\right)$ is the range resolution.

**Figure 5:**
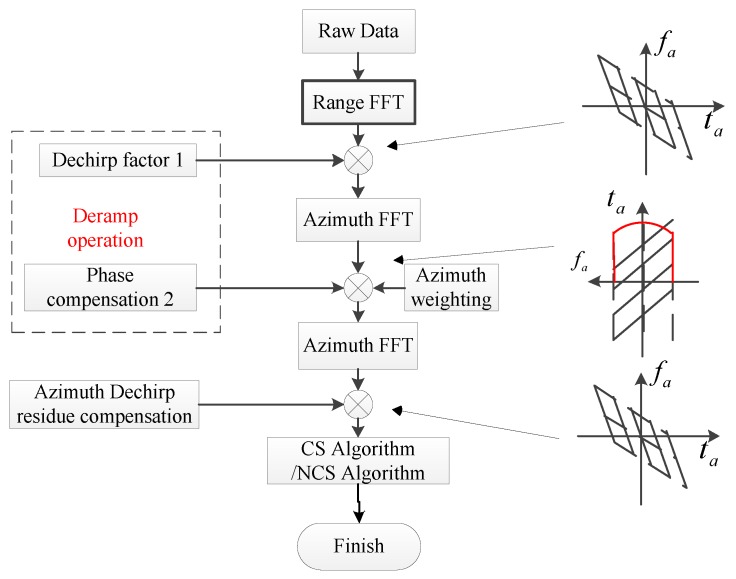
In the procedure of the algorithm of sliding-spotlight SAR imaging, the weighting method is carried out in the range frequency domain. The red line shows the window in the weighting method. The right three pictures shows the time frequency relationship in the processing steps.

The complete algorithm is shown in Figure 5. Firstly, a dechirp operation is carried out for removing the azimuth beam rotation; here, a weighting operation is applied. Secondly, the imaging algorithm, i.e., the chirp scaling algorithm [27] (pp. 206–210) [28] or nonlinear chirp scaling algorithm [29,30,31] is applied to focus the SAR image. The main difference between the presented weighting method in this article and the weighting method in [26] is in which domain the weighting operation is performed, which influences the sidelobe suppression performance, especially under the condition of high resolution.

### 3.2. The Comparison of the Former Weighting Method and Proposed Weighting Method

Up to now, a weighting method in azimuth preprocessing has been proposed, since the error of the proposed method is small, but the error of the former weighting method is great. This article is supposed to give an analysis of the error of the weighting and conduct a deramping operation in the range time domain [26], and perform weighting and conduct a deramping operation in the range frequency domain.

Actually, the deterioration of the azimuth resolution and PSLR (Peak Side Lobe Ratio) indeed changes along the varying resolution; the reason is complex and a detailed discussion is illustrated in this part.

Consider two different processing methods:

#### 3.2.1. Weighting and Conducting a Deramping Operation in the Range Time Domain

• The loss of resolution.

**Figure 6:**
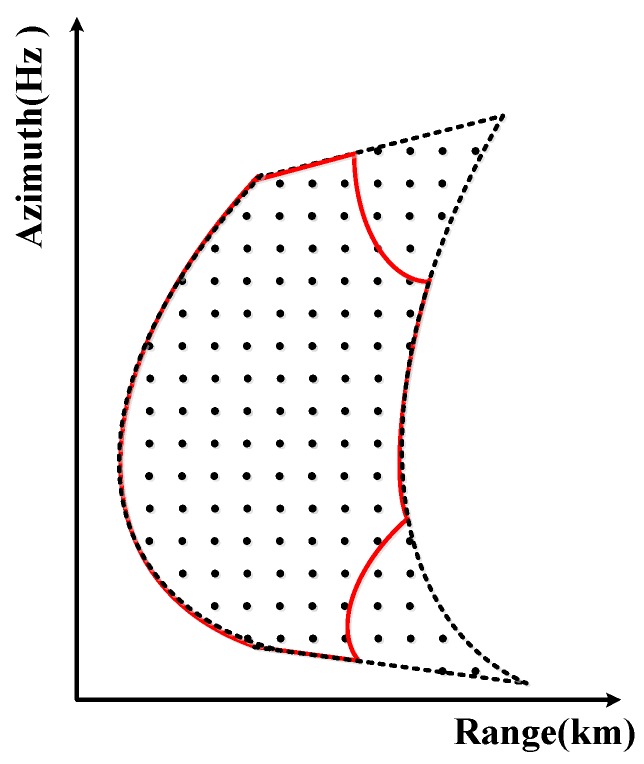
Simulated image using the former weighting method [26] with the deramping operation in the range time domain. The red line shows the residual spectrum in the range-Doppler domain after a deremping operation while using the former weighting method [26], and the dotted line shows the spectrum in the range-Doppler domain after a deremping operation without a weighting operation.

Due to the range cell migration (RCM), the amplitude of a single target in the range-Doppler domain is shown in Figure 6.

In Figure 6, the range-Doppler spectrum is lost due to weighting operation. However, notice that the updating of $f_{r}$ along the range direction is impossible, which is due to the aliasing of the range signal in the time domain. Due to the RCM, after the weighting operation [26] the residual range-Doppler spectrum is curved, as the red line shows. Finally, resolution will be lost both along the range direction and azimuth direction because of the two-dimensional spectrum losses.

• The loss of PSLR.

The azimuth PSLR and ISLR (Integrated Side Lobe Ratio) will be lost due to the wrong boundary of the weighting operation.

**Figure 7:**
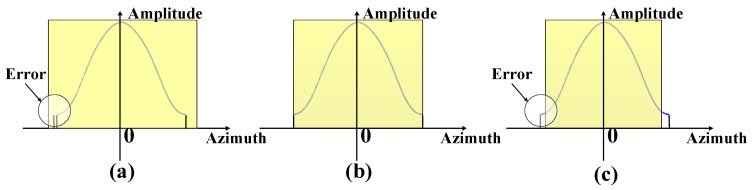
Different errors in the weighting operation. The purple line shows the window and the yellow region is the effective bandwidth that needs to be weighted. (**a**) The length of the window is shorter than the effective bandwidth; (**b**) The length of the window is equal to effective bandwidth; (**c**) The length of window is larger than the effective bandwidth.

The effect of different errors in the weighting operation is shown in Figure 7; if the length of the window is shorter than the effective bandwidth, resolution loss occurs. If the length of the window is larger than the effective bandwidth, PSLR loss occurs due to the effective window being just a part of the total window.

To sum up, the loss will be found both in the range direction and the azimuth direction, which includes resolution loss and PSLR loss. If the resolution is higher, the resolution loss and PSLR loss is higher, which is unacceptable. The quantitative analysis is too complicated and it is related with $B_{r}$, $f_{0}$, and azimuth resolution. Additionally, the necessity of the quantitative error analysis is not enough due to the former weighting method being easy to improve by weighting in the range frequency domain. Thus, the simulation results are illuminated in Section 4.

#### 3.2.2. Weighting and Conducting a Deramping Operation in the Range Frequency Domain

Conducting the weighting operation in the range frequency domain is reasonable and easy to achieve. The purpose of this part is to analyze the influence of the former bandwidth $B_{ar}\approx 2V_{s}\cdot \beta /\lambda$ and new bandwidth $B_{ar}\left(f_{r}\right)\approx 2V_{s}\cdot \beta /c\cdot \left(f_{0}+f_{r}\right)$.

• The loss of resolution.

**Figure 8:**
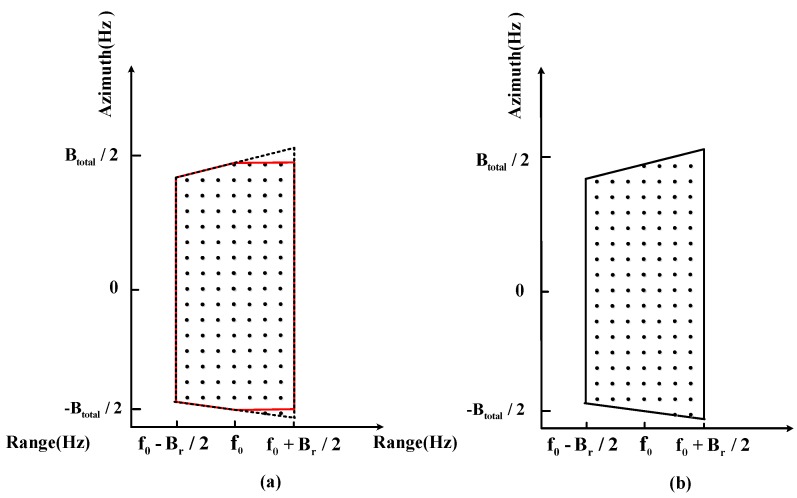
Conducting the weighting operation in the range frequency domain. The figure shows the spectrum in the two-dimensional frequency domain after a deramping operation. (**a**) Using the former weighting bandwidth$B_{ar}\approx 2V_{s}\cdot \beta /\lambda$, the red line shows the residual two-dimensional frequency spectra after the weighting operation, while the black dotted line shows the spectrum without the weighting operation. (**b**) Using the new bandwidth $B_{ar}\left(f_{r}\right)\approx 2V_{s}\cdot \beta /c\cdot \left(f_{0}+f_{r}\right)$ while updating $f_{r}$ in the range frequency domain.

In the two-dimensional frequency domains, as Figure 8 shows, RCM does not exist, while the frequency spectrum is trapezoidal. The two-dimensional frequency spectra are lost due to the weighting operation without updating $f_{r}$ along the range. However, the proposed weighting method can match the frequency spectrum perfectly and lead to no loss. Therefore, the following error analysis is about conducting the weighting operation in the range frequency domain without updating $f_{r}$.

Similarly, the loss of resolution is because of the loss of the spectrum in the two-dimensional frequency domains. Since the spectrum is trapezoidal, calculating the loss of the azimuth resolution is achievable. The parameter α is introduced, which is defined as:

(22) α=Br2f0=frot_errorfrot_ref=2Vs2cBr⋅2θT2Vscf0⋅2θT.

where α represents the ratio of the range bandwidth to the carrier frequency; this parameter can reflect the $f_{rot}$ error in the whole deramping operation and finally determines the loss of the azimuth resolution and PSLR.

**Figure 9:**
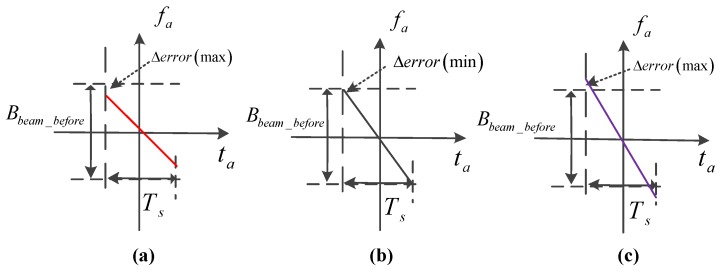
After dechirp processing the Doppler time-frequency relationship in the range frequency domain, which shows the error caused by the wideband signal without updating $f_{r}$, the max error is caused by $f_{r}=\pm B_{r}/2$. The red line shows the Doppler time-frequency relationship in (**a**) while $f_{r}=-B_{r}/2$. The purple line shows the Doppler time-frequency relationship in (**b**) while $f_{r}=B_{r}/2$. The black line shows the Doppler time-frequency relationship in (**c**) while $f_{r}=0$.

The error caused by the wideband signal without updating $f_{r}$ is shown in Figure 9, the loss of resolution is shown with dash line, and the loss of azimuth bandwidth is proportionate to $f_{rot\_error}/f_{rot\_ref}$. Moreover, the total error is the integration along the range frequency $f_{r}$, the range frequency distribution is uniform, and the error integral is expressed as:

(23) $$mean(\mathrm{error})=\frac{\frac{1}{2}\alpha \int_{0}^{\frac{1}{2}B_{r}}df_{r}}{B_{r}}=\frac{1}{4}\alpha .$$

In Figure 9, the error caused by the wideband signal means every point is inaccurate if $f_{r}=0$. This error leads to two other kinds of errors:

1. If $f_{r}<0$, as the red line shows:

$f_{rot}\left(f_{r}\right)-f_{rot}\left(0\right)=-2V_{s}\cdot \left(f_{0}+f_{r}\right)/c\cdot 2\theta /T+2V_{s}\cdot \left(f_{0}\right)/c\cdot 2\theta /T>0$, the dechirp operation exceeds the original ratio, leading to over-dechirp, and finally causes the azimuth PSLR loss, which is because the azimuth weighting window is larger than the residual bandwidth.

2. If $f_{r}>0$, as the purple line shows:

$f_{rot}\left(f_{r}\right)-f_{rot}\left(0\right)=-2V_{s}\cdot \left(f_{0}+f_{r}\right)/c\cdot 2\theta /T+2V_{s}\cdot \left(f_{0}\right)/c\cdot 2\theta /T<0$, the dechirp operation is less than the original ratio, leading to the lack of dechirp, and finally causes the azimuth resolution loss, which is because the azimuth weighting window is shorter than the residual bandwidth.

Only by updating $f_{r}$ in the weighting operation could the length of the azimuth window be controlled and, finally, can precise azimuth weighting be achieved.

• The loss of PSLR

Similarly, the azimuth PSLR and ISLR will be lost due to the wrong boundary of the weighting operation, as Figure 7 shows. The simulation results are illuminated in Section 4.

To sum up, the loss will be found both in the range direction and the azimuth direction, which includes resolution loss and PSLR loss. α is directly proportional to the resolution loss and shows a positive correlation with the PSLR loss. We further mention the weighting result of our method is close to the theoretical value, as presented in the experimental part.

## 4. Experimental Results

A number of simulations have been carried out with the resolution varying from 0.2 m to 1 m, which are illustrated as follows. The imaging algorithms are the same and a −25 dB Taylor window is adopted in the following simulations.

Consider two different processing methods:

### 4.1. Weighting and Conducting a Deramping Operation in the Range Time Domain

**Figure 10:**
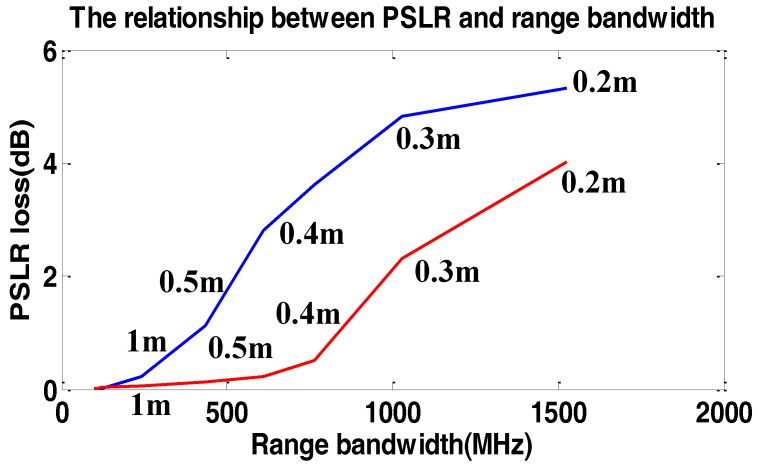
The relationship between PSLR loss and range bandwidth weighting in the range time domain. The blue line shows the PSLR loss in the azimuth direction and the red line shows the PSLR loss in the range direction. The numbers beside the line show the two-dimensional theoretic resolution.

**Figure 11:**
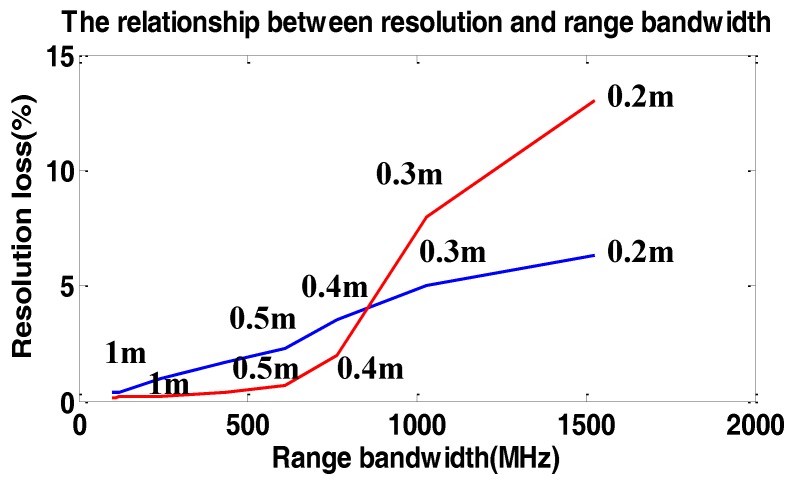
The relationship between resolution loss and range bandwidth weighting in the range time domain. The blue line shows the resolution loss in the azimuth direction and the red line shows the resolution loss in the range direction. The numbers beside the line show the two-dimensional theoretic resolution.

To show the loss of PSLR and resolution in sub-meter resolution data processing in the range time domain [26], we simulated a single target with the resolution varying from 0.2 m to 1 m. The carrier frequency $f_{0}=5.4\;\mathrm{GHz}$. The result of the simulations are shown in Figure 10 and Figure 11.

In Figure 10 and Figure 11, the loss of resolution and PSLR are both in the range and azimuth directions because of two-dimensional coupling. As we can see, the loss of resolution and PSLR are both intolerable with the increase of resolution.

We further note if the carrier frequency is increased, the loss of resolution and PSLR are both decreased.

### 4.2. Weighting and Conducting a Deramping Operation in the Range Frequency Domain

**Figure 12:**
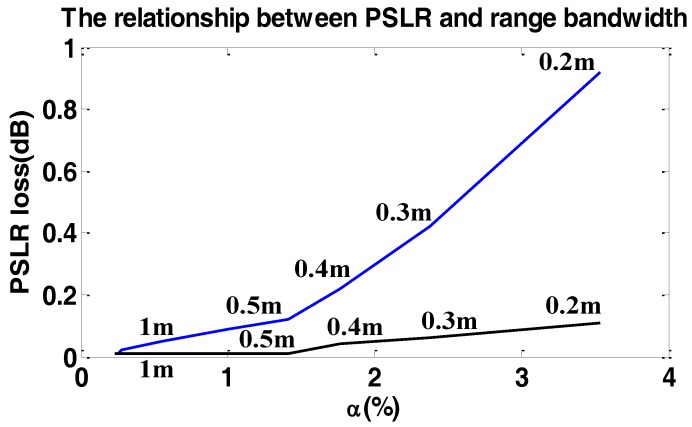
The relationship between PSLR loss and α. The blue line shows the PSLR loss in the azimuth direction without considering the variation of $f_{r}$, the black line shows the PSLR loss in the azimuth direction with considering the variation of $f_{r}$. The numbers beside the line show the two-dimensional theoretic resolution.

**Figure 13:**
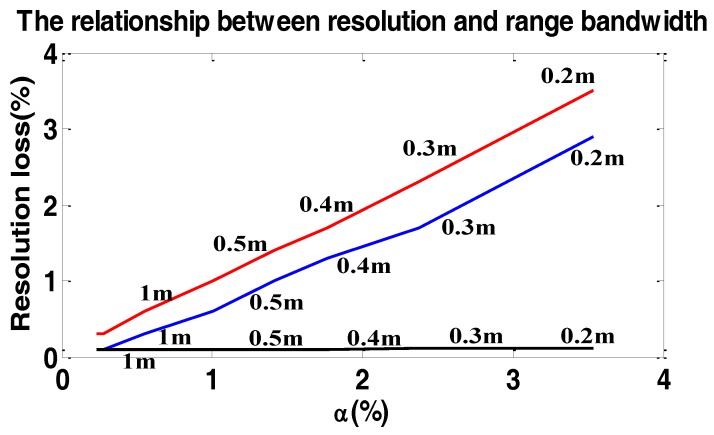
The relationship between resolution loss and α. The blue line shows the resolution loss in the azimuth direction without considering the variation of $f_{r}$. The red line shows the theoretic resolution loss without considering the variation of $f_{r}$. The black line shows the resolution loss with considering the variation of $f_{r}$. The numbers beside the line show the two-dimensional theoretic resolution.

To show the loss of PSLR and resolution in sub-meter resolution data processing in the range frequency domain, many experiments were carried out. We simulated a single target with the resolution varying from 0.2 m to 1 m. The result of the simulations are illustrated in Figure 12 and Figure 13.

In Figure 12 and Figure 13, the loss of resolution and PSLR are given in the azimuth direction. The loss in the range direction is so small that we can neglect it. As we can see, with the increase of α, the loss of resolution and PSLR are both intolerable. Additionally the theoretical value is shown by the red line, which is close to the simulated values. However, the performance of the proposed weighting method is hardly influenced by α, which means the proposed method is suitable for the high-resolution spotlight sliding method azimuth weighting.

We further mention if the carrier frequency is increased, the loss of resolution and PSLR are both decreased. Namely, the loss of resolution is inversely proportional to the carrier frequency.

### 4.3. The Simulation at 0.3 m Resolution

**Figure 14:**
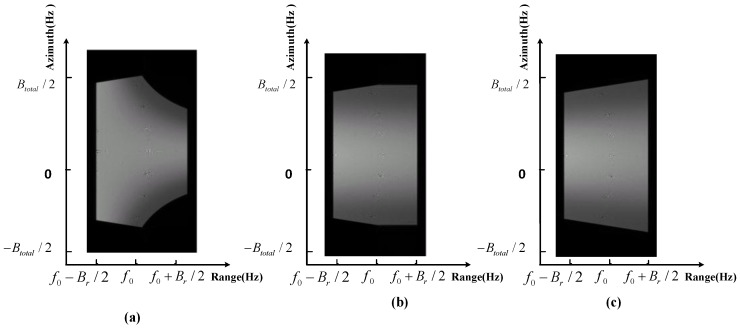
The comparison of two dimensional spectrum of a single target at 0.3 m resolution using different azimuth weighting methods. (**a**) Conducting azimuth weighting in the range time domain; (**b**) Conducting azimuth weighting in the range frequency domain without updating $f_{r}$; (**c**) The proposed weighting method in the range frequency domain with updating $f_{r}$.

In order to verify the weighting algorithm performance at high-resolution, a 0.3 m resolution sliding spotlight mode simulation is conducted as a specific example. Three weighting methods are adopted in the imaging simulation with the same system parameters as in Table 1. The imaging algorithms are all NCSA (Nonlinear Chirp Scaling Algorithm) while the difference between a, b, and c procedures in Figure 14 are the weighting method.

**Figure 15:**
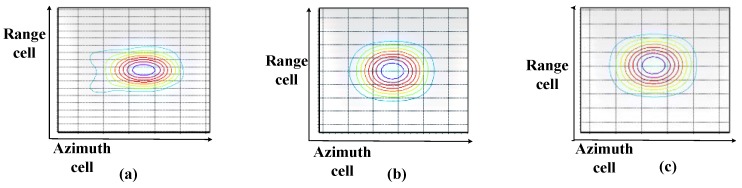
Simulated image using different window-adding methods. Contour lines are just an example to show the result. (**a**) Conducting azimuth weighting in the range time domain; (**b**) Conducting azimuth weighting in the range frequency domain without updating $f_{r}$; (**c**) The proposed weighting method in the range frequency domain with updating $f_{r}$.

Now the two-dimensional spectrum losses are shown in Figure 14. Weighting in the range time domain losses a lot of spectrum, which leads to great resolution loss. Weighting in the range frequency domain without updating $f_{r}$ also leads to some resolution losses. The proposed weighting method in the range frequency domain with updating $f_{r}$ performs well. The comparison of the three azimuth weighting method is shown in Table 2 and the contour of the three imaged point targets is shown in Figure 15.

In Figure 15 and Table 2, the losses exist both in weighting methods a and b, which are unacceptable, while the proposed weighting method performs well. The data are in the two-dimensional frequency domain after a precise deramping method and followed by 2-dimensional compensation naturally, which means the computational load of the proposed weighting method is not increased.

**Figure 16:**
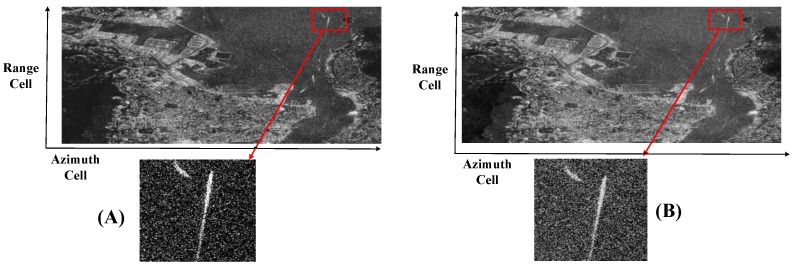
A 0.3 m resolution sliding spotlight image. (**A**) Using the proposed weighting method; (**B**) Using the azimuth weighting method in the range time domain. The extension of the area is of about 1 km × 1 km.

To compare the influence of the area target, the simulation with the same parameters in Table 1 is conducted, and the result is shown in Figure 16.

The boundary in the left picture is clearer than the right picture in Figure 16. Improving the quality of the focusing images without any extra computation burden is significant.

## 5. Conclusions

The spaceborne sliding-spotlight mode could be widely adopted for future spaceborne remote sensing. However, the wideband signal will influence its weighting performance when a two-step algorithm is adopted in the system. Based on classical azimuth preprocessing methods, carefully analysis of the signal character, and proposing the azimuth weighting method in the range frequency domain, updating $f_{r}$, then based on the weighting requirement we compare the former weighting method and the proposed weighting method with the varying resolution. Finally, the simulation results validate the presented analysis in the paper.

## Figures and Tables

**Table 1:** System parameters.

Parameter	Value
Carrier frequency	5.4 GHz
Looking angle	26°
PRF	4912 Hz
Azimuth Beam width	0.47°
Pulse bandwidth	1028 MHz
Effective velocity	7089 m/s

**Table 2:** Results of the imaging algorithm with three weighting method.

Weighting Method	Azimuth			Range		
	PSLR (dB)	ISLR (dB)	*ρ_a_* (*m*)	PSLR (dB)	ISLR (dB)	*ρ_r_* (*m*)
**a**	−20.05	−19.36	0.370	−22.62	−19.40	0.167
**b**	−24.50	−20.01	0.358	−25.11	−20.37	0.154
**c**	−24.90	−20.09	0.352	−25.26	−21.25	0.154
